# Human ADA2 Deficiency: Ten Years Later

**DOI:** 10.1007/s11882-024-01163-9

**Published:** 2024-07-06

**Authors:** Marjon Wouters, Lisa Ehlers, Mariia Dzhus, Verena Kienapfel, Giorgia Bucciol, Selket Delafontaine, Anneleen Hombrouck, Bethany Pillay, Leen Moens, Isabelle Meyts

**Affiliations:** 1 Laboratory for Inborn Errors of Immunity, Microbiology Immunology and Transplantation, KU Leuven, Louvain, Belgium; 2 Department of Pediatrics, University Hospitals Leuven, Herestraat 49, 3000 Louvain, Belgium

**Keywords:** ADA2, Stroke, Bone marrow failure, TNF Inhibition

## Abstract

**Purpose of Review:**

In this review, an update is provided on the current knowledge and pending questions about human adenosine deaminase type 2 deficiency. Patients have vasculitis, immunodeficiency and some have bone marrow failure. Although the condition was described ten years ago, the pathophysiology is incompletely understood

**Recent Findings:**

Endothelial instability due to increased proinflammatory macrophage development is key to the pathophysiology. However, the physiological role of ADA2 is a topic of debate as it is hypothesized that ADA2 fulfils an intracellular role. Increasing our knowledge is urgently needed to design better treatments for the bone marrow failure. Indeed, TNFi treatment has been successful in treating DADA2, except for the bone marrow failure.

**Summary:**

Major advances have been made in our understanding of DADA2. More research is needed into the physiological role of ADA2

## Introduction

Human adenosine deaminase type 2 (ADA2) deficiency (DADA2) is a recently described inherited autoinflammatory inborn error of immunity (IEI), characterized by systemic vasculitis with stroke, cytopenia and bone marrow failure and immunodeficiency [1]. DADA2 is caused by biallelic deleterious variants of the ADA2 gene and was first described in 2014 [2, 3]. From the first descriptions, the complexity of the phenotype and pathophysiology were apparent. Indeed, over 400 cases have been described so far, and the phenotype has expanded significantly [4–6]. Whereas the condition usually has its onset in childhood, diagnosis in adult life has been described, which highlights the need for increased awareness of this condition across disciplines and beyond pediatrics [7–9]. Using a cut-off of 75% of residual ADA2 adenosine deaminase activity in in vitro modeling of the pathogenic variants, the carrier frequency of pathogenic variants is estimated at 1:236, translating into a prevalence of 1:222,000 [10], making this a vasculitis condition that should not be neglected.

## DADA2 Clinical Phenotypes

By and large, three major phenotypes can be defined, which can all be present in a single patient. The vasculitis and/or inflammatory phenotype consists of recurrent fevers, with elevation of inflammatory markers, but also skin manifestations ranging from livedo racemosa to ulcerations and necrosis. Vasculitis also leads to stroke and central nervous system hemorrhage, next to neuropathy. Finally, end-organ vasculitis (intestinal, kidney, …) is an important manifestation and its assessment should be included in the diagnostic and routine follow-up evaluation of DADA2 patients. Nodular regenerative hyperplasia of the liver, as well as focal nodular hyperplasia and non-cirrhotic portal hypertension, a specific hepatitis and fibrosis, have been described, inviting for closer attention to the liver in patients with DADA2 [11–13]. This is outlined in the recently published DADA2 consensus statement, which provides clinicians with a practical guideline for diagnosis and management and was inspired by the DADA2 foundation [14] (Table 1).

**Table 1:** DADA2 should be in your differential in a patient presenting with one or a combination of these phenotypes

**Vasculitis and inflammation**
Recurrent fever
Livedo racemose
Cutaneous necrosis
Ulcerations, aphtjous stomatitis
Stroke: ischemic stroke and haemorrhagic stroke (deep nuclei and brain stem)
Neuropathy
Encephalitis

**Haematological manifestation**
Anemia, thrombocytopenia, neutropenia (autoimmune or due to marrow failure)
Evans syndrome, aplastic anaemia
Bone marrow failure
Lymphoproliferation benign / malignantL lymphoma T-LGL
Hemophagocytosis like episodes

**Immunodeficiency**
B cell deficiency, hypogammaglobulinemia with recurrent infections
Severe EBV, refractory warts, infections with Herpesviruses

Neurological manifestations in DADA2 present a significant clinical problem with strokes being a prominent feature carrying a high risk of fatality. In a recent review, we compiled the reported neurological events reported in DADA2 [15]. The reviewed data encompassing 628 patients reveals that 50.3% experienced at least one reported neurological event, showcasing a broad spectrum of manifestations [15]. Cerebrovascular accidents were predominant, with 77.5% of patients exhibiting clinical signs, particularly lacunar strokes in the brain stem and cerebellum and deep gray matter which account for almost 75% of stroke localizations [15]. Notably, the mean age of onset of neurological manifestation was estimated at 7.2 years, and 35.9% of those with neurological involvement had multiple stroke episodes. Beyond strokes, diverse neurological complications included neuropathies, focal deficits, ophthalmological abnormalities, convulsions, and headaches. The findings of this overview emphasize the importance of considering DADA2 in patients with 'unusual' neurological symptoms, as these can be the initial or sole manifestations in 5.7% and 0.6% of cases, respectively [15]. In a recent study of a Dutch cohort of 29 patients with DADA2, 28% of patients presented with TIA or ischemic stroke [16]. In these cases, the correct diagnosis is especially crucial since the usual antiplatelet and anticoagulant agents for the management and prevention of strokes can cause secondary hemorrhagic strokes and are contraindicated in DADA2. Therefore, stroke, encephalitis, posterior reversible encephalopathy, mononeuropathy and polyneuropathy, and Behçet’s disease-like presentations should prompt healthcare professionals to consider excluding ADA2 deficiency, especially, but not only in childhood. Additionally, neurocognitive disorders, such as autism spectrum disorder and attention deficit hyperactivity disorder, were identified in 3.5% of DADA2 patients, which suggests that the evaluation of these conditions should be part of the routine management of DADA2 [15].

Hematological and immunological manifestations are diverse in DADA2. Anemia, neutropenia, thrombocytopenia and pancytopenia are part of the DADA2 phenotype and can be either auto-immune in origin or due to bone marrow failure with marrow aplasia. Lymphoproliferation was noted in the very first reports on DADA2, and further confirmed to be present in the form of generalized lymphadenopathy and hepatosplenomegaly in up to 30% of patients [4]. Hemophagocytic lymphohistiocytosis (HLH) has been reported and can be fatal [17, 18]. Malignant lymphoproliferation has also been reported in the form of lymphoma (Non-Hodgkin, Hodgkins, and Castlemann lymphoma) [19] and T-Large granular lymphocyte (LGL) lymphoma [20]. Moreover, DADA2 patients can have a phenotype of immunodeficiency. They can experience mild infections with mild hypogammaglobulinemia, or a more impressive immunodeficient phenotype, resembling common variable immunodeficiency with recurrent sinopulmonary infections [21, 22]. Typically DADA2 patients display reduced memory B cells as well as impaired differentiation of CD4 + and CD8 + memory T cells, accelerated exhaustion/senescence, and impaired survival and granzyme production by ADA2 deficient CD8 + T cells [23]. It is unclear to what extent DADA2 patients without clinical immunodeficiency display this immunophenotype. Also, there seems to be a peculiar predisposition to protracted disease with Epstein-Barr virus (EBV). Le Voyer et al. reported on an adult with protracted EBV viremia and pure red cell aplasia. After she suffered a stroke, the diagnosis of DADA2 was made [24]. Several other papers have highlighted protracted course of EBV infection sometimes linked to malignancy [25, 26]. Also, two out of three children with DADA2 who had Hodgkin Lymphoma were EBV seropositive [19]. The impaired survival and the exhausted phenotype of the DADA2 CD8 + T cells may be in line with this [23]. Indeed, impaired T cell immunity to EBV or specific defects in T cell costimulation have been linked to increased susceptibility to severe EBV or EBV-related disease where the tight balance to control the EBV latency in B cells is lost [27]. Unraveling the intrinsic hole in immunity in DADA2 which leads to EBV susceptibility, can hopefully also aid in understanding the other unknowns in the equation.

## Diagnosis and Treatment of DADA2: In Search of the Pathophysiology

Identification of biallelic known pathogenic variants in ADA2, or of biallelic variants of uncertain significance, together with determination of severely diminished or absent ADA2 enzyme activity in the serum or plasma, is diagnostic of ADA2 deficiency [14]. The mutations span all domains of the ADA2 protein and most mutations are missense. Rarely, splice variants and intronic variants or structural variations ask for additional approaches for diagnosis [28]. Both a HPLC and spectrophotometric assays are available for diagnosis. Lee et al. have published convincing data on a potential genotype phenotype correlation, with variants resulting in absent ADA2 activity in an in vitro HEK293T transient overexpression system being linked to pure red cell aplasia and bone marrow failure phenotypes whereas variants with residual ADA2 activity correlating with the vascular phenotype [29]. Treatment is relatively straightforward for DADA2 vasculitis/vasculopathy; tumor necrosis factor alpha inhibition (TNFi) has shown to be extremely efficient in treating the vasculitis and in preventing strokes [30, 31]. Unfortunately, the immunodeficiency and hematological manifestations are less amenable to TNFi treatment. Although it has been claimed that TNFi restores B cells and antibody deficiency, this can no longer be sustained [23]. Moreover, the cytopenia and bone marrow failure particularly are difficult to treat. Combinations of growth factor therapy and cyclosporine together with TNF inhibition have resulted in short-lasting recoveries. However, mostly, these patients need hematopoietic stem cell transplantation (HSCT), which in DADA2 is particularly problematic with an undue need for second transplants because of graft failure [12, 13, 32]. A more profound T cell depletion may aid in reducing the need for sequential HSCT although this is yet to be proven. A long-term outcome study will follow soon.

Although the condition has been described almost 10 years ago and although TNFi therapy is efficient for many DADA2 patients with vasculitis, treatment gaps are evident: breakthrough inflammation does occur and auto-antibody development to TNFi is a threat. In addition, for patients with bone marrow marrow failure, there is an urgent medical need for a targeted treatment. Such targeted approach is hampered by the fact that the pathogenesis of DADA2 and functional significance of ADA2 protein is not fully understood.

The prevailing hypothesis is that ADA2 deficiency leads to skewed M1 macrophage development, which in turn results in disturbed endothelial integrity [33]. Although TNF is one of the downstream key cytokines in the inflammatory cascade led by NF-kB and IFN, many questions remain, which the DADA2 scientific and medical community will need to solve in order for a more targeted treatment of DADA2 [33]. Although the monocytes are the cells that express ADA2 most abundantly, a role in the pathophysiology has been proposed for neutrophils and endothelial cells (unpublished observation Marjon Wouters) [34, 35]. Finally, a high risk of cancer has been reported in the Dutch cohort, and three patients in the authors’ cohort have suffered various malignancies, raising the question whether there is indeed an increased risk of malignancy [17]. It remains to be determined whether this is intrinsic or secondary to treatment and longstanding inflammation.

## Type I and II Interferons in DADA2: DADA2 as an Interferonopathy?

Strong type I and type II interferon (IFN) gene expression signatures have been described in DADA2 patients. An upregulation of type I IFN gene expression in DADA2 patients was first reported in 2014 by Belot et al. [36]. Based on the clinical overlap between type I interferonopathy *SAMHD1* Aicardi-Goutières syndrome (AGS) and DADA2, the interferon signature in the peripheral blood of two DADA2 patients was investigated. An upregulation of IFN-stimulated genes (ISGs) was observed in both patients [36]. Moreover, Skrabl-Baumgartner et al. showed an increased expression of ISGs was present in the peripheral blood of two brothers. As they presented with inflammatory systemic vasculitis affecting brain and skin, AGS was suspected. However, whole exome sequencing revealed biallelic mutations in the *ADA2* gene [37]. Insalaco et al. observed an increased interferon score (IS) in four DADA2 patients in a cohort of five. In addition, this increased gene signature decreased markedly after starting etanercept. Comparable plasma levels of CXCL9 and CXCL10 were observed in healthy controls and DADA2 patients, implying no activation of type II IFN pathways. Furthermore, a correlation was observed between disease severity as depicted by CRP and the degree of elevation of the interferon score (IS). This hinted the possible utility of the IS as biomarker to assess disease activity, severity and treatment response [38]. However a cohort study of 4 Japanese DADA2 patients opposed the use of the IS as a possible biomarker. They indeed found an increased IS in DADA2 patients compared to healthy controls, albeit not as high as those observed in type 1 interferonopathies AGS. Moreover the IS did not change after treatment with TNFi. More strikingly, multi-omics analysis revealed an upregulation of type II IFN related genes. This upregulated expression remained significantly higher compared to healthy controls. However, when type II IFN related gene expression was compared in DADA2 patients before and after treatment, levels were lower after starting anti-TNF-alfa [39]. Chen et al. showed an upregulation of both type I as well as type II interferon signaling pathways in the peripheral blood of 14 DADA2 patients. However, upregulation of type I IFN signaling was generally not as high as observed in other type I interferonopathies. The significance of this remains ambiguous since this upregulation is observed across different clinical presentations [40]. In conclusion, there is upregulation of type I and type II IFN in DADA2, but the classification of this condition as a type I interferonopathy was probably less appropriate. While searching for an optimal serum biomarker, we have designed a DADA2 damage and activity score to allow for longitudinal monitoring of disease manifestations [41] (Table 2).

**Table 2:** Non-exhaustive overview of type I and type II IFN signature measurement in DADA2 patients

Number of patients	Genotype	Phenotype	Type I IFN signature (number of measurements)	Response to therapy	Type IIF IFN	Response to therapy	Ref
2	1:c.506G > A,c.578C > T; (p.Pro193Leu, p.Arg169Gln) 2:homozygous c.139G > C/ (p.Gly47Arg)	Vasculitis -stroke Vasculitis – stroke	Increased (3) Increased (1)	ND	ND	ND	[35]*
2	1:c.139G > C, c.1223G > A (p.Gly47Arg, p-Cys408Tyr) 2:c.139G > C,c.1223G > A (p.Gly47Arg, p-Cys408Tyr)	Vasculitis – stroke Vasculitis – stroke	Increased (1) Increased (1)	ND	ND	ND	[36]**
5	1:c.144de1G, p.Arg49Glyfs*4, c.1078A > G, p. Thr360Ala 2:c.1358A > G mutation, p.Tyr453Cys (rs376785840), 3,4:c.746 T > C,c.1031C > T, (p.Leu249Pro, p.Pro344Leu) 5.c.1358A > G, c.133C > T (p.Tyr453Cys,p.Arg45Trp)	Vasculitis Vasculitis Vasculitis – PRES One episode of fever Vasculitis	Increased in 4/5 (1)	Response to etanercept in 4/4	NL plasma CXCL9 and CXCL10	ND	[37]*
4/8	c.982G > A, p.Glu328Lys -Unknown c.982G > A, p.Glu328Lys -Unknown c.984G > C, c.706_ 708TACdel (p.Glu328Asp,p.Tyr236del) p.Glu328Asp (c.984G > C) p.Tyr236del (c.706_ 708TACdel)	Vasculitis– stroke Vasculitis Vasculitis PRCA	Increased	No	Increased	Yes	[38]***
14/33			Elevated (also in 3 asymptomatic individuals)		Elevated (also in 3 asymptomatic individuals)		[39]***

Methods: *6 gene panel, **7 gene panel, ***transcriptomics

*ND *not discussed in the report, *PRCA *pure red cell aplasia, *PRES* posterior reversible encephalitis syndrome, *NL* normal

## Looking Inside: Characteristics of the ADA2 Glycoprotein

Human adenosine deaminase 2 (ADA2) was first analyzed in spleen extracts from patients with ADA-SCID in 1978 [42]. The researchers identified a protein accounting for remnant adenosine deaminase activity in the absence of functional ADA1. This second adenosine deaminase was unaffected by the ADA1-inhibitor erythro-9-(2-hydroxy-3-nonly)adenine (EHNA), had a lower pH optimum at 6.5 and a Michaelis constant of around 2 mM. Many years later, this protein was found to be encoded by a gene on chromosome 22 in the region affected in patients with cat eye syndrome, hence the original gene name *CECR1* (cat eye syndrome critical region protein 1) [43].

While the catalytic regions of ADA1 and ADA2 show strong structural similarities, the protein characteristics of the two isoenzymes differ greatly [44]. ADA1 is a 40-kD monomer with a high affinity for adenosine (Km = 40 µM). It is a cytosolic protein but can localize to the cell surface as ecto-ADA1 where it forms a complex with CD26 [45]. ADA2, on the other hand, has several features characteristic of proteins of the secretory pathway: an N-terminal signal peptide, four N-glycosylation sites and a putative receptor binding domain [44]. ADA2 can form homodimers and the dimerization site mutant p.W362G shows decreased catalytic deaminase activity [44].

N-glycosylation is crucial to ensure the structural integrity of ADA2 [46, 47]. Removing N-glycans enzymatically after successful folding and secretion of the protein does not affect its deaminase function [47]. Inhibition of N-glycosylation in vitro, on the other hand, leads to accumulation of ADA2 in the endoplasmic reticulum and therefore impairs secretion and enzymatic activity [46]. Endoplasmic reticulum retention with reduced secretion and deaminase activity are characteristics mirrored by mutant ADA2 protein expressed in HEK293T cells transfected with pathogenic *ADA2* variants [40]. Consequently, ADA2 has long been believed to be responsible for adenosine deamination in the extracellular space and excess extracellular adenosine has been proposed to be the mechanism underlying inflammation in ADA2 deficiency (DADA2) [34, 35]. The physiological relevance of deamination of extracellular adenosine by ADA2 is however debated.

Given the low affinity of ADA2 for adenosine, the amount of extracellular adenosine metabolized by ADA2 at physiological plasma concentrations of adenosine (10–20 nM) should be very low in the presence of functional ADA1 [48, 49]. Even in an inflammatory environment with increased levels of adenosine, ecto-ADA1 stills perform most of the extracellular adenosine deamination [48]. Moreover, even in the absence of ecto-ADA1, equilibrative nucleoside transporters should allow surplus extracellular adenosine due to ADA2 deficiency to be catabolized by intracellular ADA1 [50].

As a result, DADA2 research is starting to move away from the extracellular space. Greiner-Tollersrud and colleagues have described the lysosomal localisation of ADA2 [51]. They reported that the glycan pattern of ADA2 was reminiscent of lysosomal proteins and showed that ADA2 could function as a DNase. DNase activity correlated with the protein’s deaminase activity and was impaired in pathogenic ADA2 protein variants. The researchers showed abnormal DNA sensing in ADA2-deficient cells, thus proposing that cell-intrinsic mechanisms underlie inflammation in DADA2.

Recently, our group identified an exclusively intracellular hypoglycosylated low-molecular-weight (LMW) form of ADA2 in human monocyte-derived macrophages from healthy controls [52]. We propose that this form is generated from fully glycosylated high-molecular-weight ADA2 by glycan trimming and localizes to the cytosol and lysosomes. LMW-ADA2 was absent in macrophages derived from DADA2 patients’ monocytes and in HEK293T cells transfected with pathogenic *ADA2* variants. These findings are consistent across the pathogenic variants with residual protein expression. We hypothesize that LMW-ADA2 might be exerting an intracellular function that is absent in ADA2-deficient cells.

In summary, a deeper look inside the cell into the intracellular function of ADA2 is warranted to move DADA2 research forward and to allow for targeted therapies for those disease manifestations not responding to the current standard of care approaches.

## Additional Questions: the GATA2 ADA2 Masquerade

ADA2 and the transcription factor GATA2 (GATA Binding Protein 2) seem to be pivotal genes involved in immune regulation and adequate hematopoiesis [53]. Deficiencies in both these genes result in immunodeficiency disorders. In 2016, a patient with ADA2 deficiency was initially diagnosed as GATA2 deficient due to a clinical presentation reminiscent of GATA2 deficiency. However, whole exome sequencing unveiled biallelic mutations in ADA2 [54]. In addition, several patients referred for severe warts, suspect for GATA2 deficiency, ultimately had DADA2 [55]. Finally, rheumatological manifestations are found in 17.8% of patients with GATA2 deficiency [56].

Despite their distinct molecular functions, there seems to be a considerable phenotypical overlap between ADA2 and GATA2. GATA2 patients exhibited lower ADA2 enzyme activity without ADA2 mutations (unpublished observation). Furthermore, unpublished data on *ADA2* and *GATA2* RNA levels in patients and controls demonstrate downregulation of both genes in patie nt cohorts, suggesting an influence on RNA expression. Since GATA2 is a complex transcription factor, it is likely that it also influences the expression of *ADA2*. We are further investigating the connection between these two complex IEIs as we believe this can give us insight into the complex pathophysiology of the bone marrow failure in DADA2.

## Conclusion

DADA2, a complex autoinflammatory condition has been described almost 10 years ago. Thanks to orchestrated efforts of scientists and physicians over the world, brough together by the DADA2 foundation, we have stridden fast forward to learning how to treat patients with DADA2. Now efforts are needed to unveil the last riddles of the pathophysiology of DADA2 to allow for targeted treatment for patients with bone marrow failure in the context of DADA2.

## Data Availability

No datasets were generated or analysed during the current study.

## References

[1] Tangye SG, Al-Herz W, Bousfiha A, Cunningham-Rundles C, Franco JL, Holland SM, et al. Human inborn errors of immunity: 2022 update on the classification from the international union of immunological societies expert committee. J Clin Immunol. 2022;42(7):1473–507. 35748970 10.1007/s10875-022-01289-3 PMC9244088

[2] Zhou Q, Yang D, Ombrello AK, Zavialov AV, Toro C, Zavialov AV, et al. Early-onset stroke and vasculopathy associated with mutations in ADA2. N Engl J Med. 2014;370(10):911–20. 24552284 10.1056/NEJMoa1307361 PMC4193683

[3] Navon Elkan P, Pierce SB, Segel R, Walsh T, Barash J, Padeh S, et al. Mutant adenosine deaminase 2 in a polyarteritis nodosa vasculopathy. N Engl J Med. 2014;370(10):921–31. 24552285 10.1056/NEJMoa1307362

[4] Meyts I, Aksentijevich I. Deficiency of adenosine deaminase 2 (DADA2): Updates on the phenotype, genetics, pathogenesis, and treatment. J Clin Immunol. 2018;38(5):569–78. 29951947 10.1007/s10875-018-0525-8 PMC6061100

[5] Sahin S, Adrovic A, Kasapcopur O. A monogenic autoinflammatory disease with fatal vasculitis: deficiency of adenosine deaminase 2. Curr Opin Rheumatol. 2020;32(1):3–14. 31599797 10.1097/BOR.0000000000000669

[6] Moens L, Hershfield M, Arts K, Aksentijevich I, Meyts I. Human adenosine deaminase 2 deficiency: A multi-faceted inborn error of immunity. Immunol Rev. 2019;287(1):62–72. 30565235 10.1111/imr.12722

[7] Betrains A, Staels F, Moens L, Delafontaine S, Hershfield MS, Blockmans D, et al. Diagnosis of deficiency of adenosine deaminase type 2 in adulthood. Scand J Rheumatol. 2021;50(6):493–6. 33627040 10.1080/03009742.2021.1881156

[8] Rama M, Duflos C, Melki I, Bessis D, Bonhomme A, Martin H, et al. A decision tree for the genetic diagnosis of deficiency of adenosine deaminase 2 (DADA2): a French reference centres experience. Eur J Hum Genet. EJHG. 2018;26(7):960–71. 29681619 10.1038/s41431-018-0130-6 PMC6018671

[9] Fayand A, Sarrabay G, Belot A, Hentgen V, Kone-Paut I, Grateau G, et al. Multiple facets of ADA2 deficiency: Vasculitis, auto-inflammatory disease and immunodeficiency: A literature review of 135 cases from literature. Rev Med Interne. 2017;39(4):297–306. 29273180 10.1016/j.revmed.2017.11.006

[10] Jee H, Huang Z, Baxter S, Huang Y, Taylor ML, Henderson LA, et al. Comprehensive analysis of ADA2 genetic variants and estimation of carrier frequency driven by a function-based approach. J Allergy Clin Immunol. 2022;149(1):379–87. 34004258 10.1016/j.jaci.2021.04.034 PMC8591146

[11] Barron KS, Aksentijevich I, Deuitch NT, Stone DL, Hoffmann P, Videgar-Laird R, et al. The spectrum of the deficiency of adenosine deaminase 2: An observational analysis of a 60 patient cohort. Front Immunol. 2021;12:811473. 35095905 10.3389/fimmu.2021.811473 PMC8790931

[12] Hashem H, Bucciol G, Ozen S, Unal S, Bozkaya IO, Akarsu N, et al. Hematopoietic cell transplantation cures adenosine deaminase 2 deficiency: Report on 30 patients. J Clin Immunol. 2021;41(7):1633–47. 34324127 10.1007/s10875-021-01098-0 PMC8452581

[13] Hashem H, Dimitrova D, Meyts I. Allogeneic hematopoietic cell transplantation for patients with deficiency of adenosine deaminase 2 (DADA2): Approaches. Obstacles and Special Considerations Front Immunol. 2022;13:932385. 35911698 10.3389/fimmu.2022.932385 PMC9336546

[14] Lee PY, Davidson BA, Abraham RS, Alter B, Arostegui JI, Bell K, et al. Evaluation and Management of Deficiency of Adenosine Deaminase 2: An International Consensus Statement. JAMA Netw Open. 2023;6(5): e2315894. 37256629 10.1001/jamanetworkopen.2023.15894

[15] Dzhus M, Ehlers L, Wouters M, Jansen K, Schrijvers R, De Somer L, et al. A narrative review of the neurological manifestations of human adenosine deaminase 2 deficiency. J Clin Immunol. 2023;43(8):1916–26. 37548813 10.1007/s10875-023-01555-y PMC10661818

[16] Verschoof MA, van Meenen LCC, Andriessen MVE, Brinkman DMC, Kamphuis S, Kuijpers TW, et al. Neurological phenotype of adenosine deaminase 2 deficient patients: a cohort study. Eur J Neurol. 2024;31(1): e16043. 37584090 10.1111/ene.16043 PMC11235617

[17] Andriessen MVE, Legger GE, Bredius RGM, van Gijn ME, Hak AE, Muller P, et al. Clinical symptoms, laboratory parameters and long-term follow-up in a national DADA2 cohort. J Clin Immunol. 2023;43(7):1581–96. 37277582 10.1007/s10875-023-01521-8 PMC10499949

[18] Drago E, Garbarino F, Signa S, Grossi A, Schena F, Penco F, et al. Case Report: Susceptibility to viral infections and secondary hemophagocytic lymphohistiocytosis responsive to intravenous immunoglobulin as primary manifestations of adenosine deaminase 2 deficiency. Front Immunol. 2022;13: 937108. 36159847 10.3389/fimmu.2022.937108 PMC9503826

[19] Alabbas F, Elyamany G, Alsharif O, Hershfield M, Meyts I. Childhood hodgkin lymphoma: Think DADA2. J Clin Immunol. 2019;39(1):26–9. 30644014 10.1007/s10875-019-0590-7

[20] Trotta L, Martelius T, Siitonen T, Hautala T, Hamalainen S, Juntti H, et al. ADA2 deficiency: Clonal lymphoproliferation in a subset of patients. J Allergy Clin Immunol. 2018;141:1534–1537.e8. 29391253 10.1016/j.jaci.2018.01.012

[21] Schepp J, Bulashevska A, Mannhardt-Laakmann W, Cao H, Yang F, Seidl M, et al. Deficiency of adenosine deaminase 2 causes antibody deficiency. J Clin Immunol. 2016;36(3):179–86. 26922074 10.1007/s10875-016-0245-x

[22] Schepp J, Proietti M, Frede N, Buchta M, Hubscher K, Rojas Restrepo J, et al. Screening of 181 patients with antibody deficiency for deficiency of adenosine deaminase 2 sheds new light on the disease in adulthood. Arthritis Rheumatol. 2017;69(8):1689–700. 28493328 10.1002/art.40147

[23] Yap JY, Moens L, Lin M-W, Kane A, Kelleher A, Toong C, et al. Intrinsic defects in B cell development and differentiation, T cell exhaustion and altered unconventional T cell generation characterize human adenosine deaminase type 2 deficiency. J Clin Immunol. 2021;41(8):1915–35. 34657246 10.1007/s10875-021-01141-0 PMC8604888

[24] Le Voyer T, Boutboul D, Ledoux-Pilon A, de Fontbrune FS, Boursier G, Latour S, et al. Late-onset EBV susceptibility and refractory pure red cell aplasia revealing DADA2. J Clin Immunol. 2020;40(6):948–53. 32643137 10.1007/s10875-020-00812-8

[25] Brooks JP, Rice AJ, Ji W, Lanahan SM, Konstantino M, Dara J, et al. Uncontrolled epstein-barr virus as an atypical presentation of deficiency in ADA2 (DADA2). J Clin Immunol. 2021;41(3):680–3. 33394316 10.1007/s10875-020-00940-1

[26] Staples E, Simeoni I, Stephens JC, Allen HL, BioResource N, Wright P, et al. ADA2 deficiency complicated by EBV-driven lymphoproliferative disease. Clinical immunology (Orlando, Fla). 2020;215: 108443. 32353633 10.1016/j.clim.2020.108443 PMC7306156

[27] Tangye SG, Latour S. Primary immunodeficiencies reveal the molecular requirements for effective host defense against EBV infection. Blood. 2020;135(9):644–55. 31942615 10.1182/blood.2019000928

[28] Ehlers L, Bucciol G, team KUL-UD, Beysen D, Meyts I. ADA2 Deficiency mimicking acute disseminated encephalomyelitis. J Clin Immunol. 2023;43(3):536–9. 36472692 10.1007/s10875-022-01413-3 PMC9957855

[29] Lee PY, Kellner ES, Huang Y, Furutani E, Huang Z, Bainter W, et al. Genotype and functional correlates of disease phenotype in deficiency of adenosine deaminase 2 (DADA2). J Allergy Clin Immunol. 2020;145(6):1664-72e10. 31945408 10.1016/j.jaci.2019.12.908 PMC7282972

[30] Ombrello AK, Qin J, Hoffmann PM, Kumar P, Stone D, Jones A, et al. Treatment strategies for deficiency of adenosine deaminase 2. N Engl J Med. 2019;380(16):1582–4. 30995379 10.1056/NEJMc1801927 PMC7372950

[31] Ombrello AK BK, Hoffmann P, Toro C, Stone DL, Pinto-Patarroyo G, Jones A, Romeo T, Soldatos A, Zhou Q, Deuitch N, Qin J, Aksentijevich I, Kastner DL. Deficiency of adenosine deaminase type 2 (DADA2)—results of anti-TNF treatment in a cohort of patients with a history of stroke. Arthritis Rheumatol. 2016;68(S10).

[32] Hashem H, Kumar AR, Muller I, Babor F, Bredius R, Dalal J, et al. Hematopoietic stem cell transplantation rescues the hematological, immunological, and vascular phenotype in DADA2. Blood. 2017;130(24):2682–8. 28974505 10.1182/blood-2017-07-798660 PMC5731089

[33] Lee PY, Aksentijevich I, Zhou Q. Mechanisms of vascular inflammation in deficiency of adenosine deaminase 2 (DADA2). Semin Immunopathol. 2022;44(3):269–80. 35178658 10.1007/s00281-022-00918-8

[34] Dhanwani R, Takahashi M, Mathews IT, Lenzi C, Romanov A, Watrous JD, et al. Cellular sensing of extracellular purine nucleosides triggers an innate IFN-beta response. Sci Adv. 2020;6(30):eaba3688. 32743071 10.1126/sciadv.aba3688 PMC7375821

[35] Carmona-Rivera C, Khaznadar SS, Shwin KW, Irizarry-Caro JA, O’Neil LJ, Liu Y, et al. Deficiency of adenosine deaminase 2 triggers adenosine-mediated NETosis and TNF production in patients with DADA2. Blood. 2019;134(4):395–406. 31015188 10.1182/blood.2018892752 PMC6659253

[36] Belot A, Wassmer E, Twilt M, Lega JC, Zeef LA, Oojageer A, et al. Mutations in CECR1 associated with a neutrophil signature in peripheral blood. Pediatr Rheumatol Online J. 2014;12:44. 25278816 10.1186/1546-0096-12-44 PMC4181355

[37] Insalaco A, Moneta GM, Pardeo M, Caiello I, Messia V, Bracaglia C, et al. Variable clinical phenotypes and relation of interferon signature with disease activity in ADA2 deficiency. J Rheumatol. 2019;46(5):523–6. 30647181 10.3899/jrheum.180045

[38] Skrabl-Baumgartner A, Plecko B, Schmidt WM, Konig N, Hershfield M, Gruber-Sedlmayr U, et al. Autoimmune phenotype with type I interferon signature in two brothers with ADA2 deficiency carrying a novel CECR1 mutation. Pediatr Rheumatol Online J. 2017;15(1):67. 28830446 10.1186/s12969-017-0193-x PMC5568374

[39] Nihira H, Izawa K, Ito M, Umebayashi H, Okano T, Kajikawa S, et al. Detailed analysis of Japanese patients with adenosine deaminase 2 deficiency reveals characteristic elevation of type II interferon signature and STAT1 hyperactivation. J Allergy Clin Immunol. 2021;148(2):550–62. 33529688 10.1016/j.jaci.2021.01.018

[40] Chen L, Mamutova A, Kozlova A, Latysheva E, Evgeny F, Latysheva T, et al. Comparison of disease phenotypes and mechanistic insight on causal variants in patients with DADA2. J Allergy Clin Immunol. 2023;152(3):771–82. 37150360 10.1016/j.jaci.2023.04.014

[41] Bucciol G, Ombrello AK, Chambers EP, Meyts I. Proposal for a Disease Activity Score and Disease Damage Score for ADA2 Deficiency: the DADA2AI and DADA2DI. J Clin Immunol. 2023;44(1):25. 38129740 10.1007/s10875-023-01638-w PMC10739542

[42] Schrader WP, Pollara B, Meuwissen HJ. Characterization of the residual adenosine deaminating activity in the spleen of a patient with combined immunodeficiency disease and adenosine deaminase deficiency. Proc Natl Acad Sci USA. 1978;75(1):446–50. 24216 10.1073/pnas.75.1.446 PMC411266

[43] Riazi MA, Brinkman-Mills P, Nguyen T, Pan H, Phan S, Ying F, et al. The human homolog of insect-derived growth factor, CECR1, is a candidate gene for features of cat eye syndrome. Genomics. 2000;64(3):277–85. 10756095 10.1006/geno.1999.6099

[44] Zavialov AV, Yu X, Spillmann D, Lauvau G, Zavialov AV. Structural basis for the growth factor activity of human adenosine deaminase ADA2. J Biol Chem. 2010;285(16):12367–77. 20147294 10.1074/jbc.M109.083527 PMC2852975

[45] Franco R, Casado V, Ciruela F, Saura C, Mallol J, Canela EI, et al. Cell surface adenosine deaminase: much more than an ectoenzyme. Prog Neurobiol. 1997;52(4):283–94. 9247966 10.1016/S0301-0082(97)00013-0

[46] Lee PY, Huang Y, Zhou Q, Schnappauf O, Hershfield MS, Li Y, et al. Disrupted N-linked glycosylation as a disease mechanism in deficiency of ADA2. J Allergy Clin Immunol. 2018;142(4):1363–1365.e8. 29936104 10.1016/j.jaci.2018.05.038 PMC6175612

[47] Ito M, Maejima Y, Nishimura K, Nakae Y, Ono A, Iwaki-Egawa S. A role for N-glycosylation in active adenosine deaminase 2 production. Biochim Biophys Acta Gen Subj. 2022;1866(12): 130237. 36029899 10.1016/j.bbagen.2022.130237

[48] Tarrant TK, Kelly SJ, Hershfield MS. Elucidating the pathogenesis of adenosine deaminase 2 deficiency: current status and unmet needs. Expert Opin Orphan Drugs. 2021;9(11–12):257–64. 10.1080/21678707.2021.2050367

[49] Lofgren L, Pehrsson S, Hagglund G, Tjellstrom H, Nylander S. Accurate measurement of endogenous adenosine in human blood. PLoS ONE. 2018;13(10): e0205707. 30359421 10.1371/journal.pone.0205707 PMC6201894

[50] Baldwin SA, Beal PR, Yao SY, King AE, Cass CE, Young JD. The equilibrative nucleoside transporter family, SLC29. Pflugers Arch. 2004;447(5):735–43. 12838422 10.1007/s00424-003-1103-2

[51] Greiner-Tollersrud OK, Boehler V, Bartok E, Krausz M, Polyzou A, Schepp J, et al. ADA2 is a lysosomal DNase regulating the type-I interferon response. 2020. BioRxiv. 10.1101/2020.06.21.162990.

[52] Ehlers L, Hombrouck A, Wouters M, Pillay B, Delafontaine S, Bucciol G, et al. Human ADA2 deficiency is characterized by the absence of an intracellular hypoglycosylated form of adenosine deaminase 2 BioRXiv bioRxiv 2023. 10.25.5640372023 [updated 2023].

[53] Spinner MA, Sanchez LA, Hsu AP, Shaw PA, Zerbe CS, Calvo KR, et al. GATA2 deficiency: a protean disorder of hematopoiesis, lymphatics, and immunity. Blood. 2014;123(6):809–21. 24227816 10.1182/blood-2013-07-515528 PMC3916876

[54] Hsu AP, West RR, Calvo KR, Cueller-Rodriguez J, Parta M, Kelly SJ, et al. Adenosine deaminase type 2 deficiency masquerading as GATA2 deficiency Successful hematopoietic stem cell transplantation. J Allergy Clin Immunol. 2016;138(2):628-30e2. 27130863 10.1016/j.jaci.2016.03.016 PMC4975951

[55] Arts K, Bergerson JRE, Ombrello AK, Similuk M, Oler AJ, Agharahimi A, et al. Warts and DADA2: a Mere Coincidence? J Clin Immunol. 2018;38(8):836–43. 30386947 10.1007/s10875-018-0565-0

[56] Amarnani AA, Poladian KR, Marciano BE, Daub JR, Williams SG, Livinski AA, et al. A Panoply of Rheumatological Manifestations in Patients with GATA2 Deficiency. Sci Rep. 2020;10(1):8305. 32433473 10.1038/s41598-020-64852-1 PMC7239896

